# The use of extracorporeal CO_2_ removal in acute respiratory failure

**DOI:** 10.1186/s13613-021-00824-6

**Published:** 2021-03-11

**Authors:** Raphaël Giraud, Carlo Banfi, Benjamin Assouline, Amandine De Charrière, Maurizio Cecconi, Karim Bendjelid

**Affiliations:** 1 Intensive Care Unit, Geneva University Hospitals, 4, Rue Gabrielle Perret-Gentil, 1205 Geneva, Switzerland; 2 University of Milan, Gruppo Ospedaliero San Donato, Milan, Italy; 3 Department of Cardio-Thoracic Surgery, Istituto Clinico Sant’Ambrogio, Milan, Italy; 4 Humanitas Clinical and Research Center, IRCCS, via Manzoni 56, Rozzano, Italy; 5 Department of Biomedical Sciences, Humanitas University, via Rita Levi Montalcini, Pieve Emanuele, 20090 Milan, Italy; 6 Faculty of Medicine, University of Geneva, Geneva, Switzerland; 7 Geneva Hemodynamic Research Group, Geneva, Switzerland

**Keywords:** Extracorporeal carbon dioxide removal, ECCO_2_R, Hypercapnia, Respiratory acidosis, ARDS, COPD

## Abstract

**Background:**

Chronic obstructive pulmonary disease (COPD) exacerbation and protective mechanical ventilation of acute respiratory distress syndrome (ARDS) patients induce hypercapnic respiratory acidosis.

**Main text:**

Extracorporeal carbon dioxide removal (ECCO_2_R) aims to eliminate blood CO_2_ to fight against the adverse effects of hypercapnia and related acidosis. Hypercapnia has deleterious extrapulmonary consequences, particularly for the brain. In addition, in the lung, hypercapnia leads to: lower pH, pulmonary vasoconstriction, increases in right ventricular afterload, acute cor pulmonale. Moreover, hypercapnic acidosis may further damage the lungs by increasing both nitric oxide production and inflammation and altering alveolar epithelial cells. During an exacerbation of COPD, relieving the native lungs of at least a portion of the CO_2_ could potentially reduce the patient's respiratory work, Instead of mechanically increasing alveolar ventilation with MV in an already hyperinflated lung to increase CO_2_ removal, the use of ECCO_2_R may allow a decrease in respiratory volume and respiratory rate, resulting in improvement of lung mechanic. Thus, the use of ECCO_2_R may prevent noninvasive ventilation failure and allow intubated patients to be weaned off mechanical ventilation. In ARDS patients, ECCO_2_R may be used to promote an ultraprotective ventilation in allowing to lower tidal volume, plateau (Pplat) and driving pressures, parameters that have identified as a major risk factors for mortality. However, although ECCO_2_R appears to be effective in improving gas exchange and possibly in reducing the rate of endotracheal intubation and allowing more protective ventilation, its use may have pulmonary and hemodynamic consequences and may be associated with complications.

**Conclusion:**

In selected patients, ECCO_2_R may be a promising adjunctive therapeutic strategy for the management of patients with severe COPD exacerbation and for the establishment of protective or ultraprotective ventilation in patients with ARDS without prognosis-threatening hypoxemia.

## Background

Extracorporeal carbon dioxide removal (ECCO_2_R) is a technique whose objective is the decarboxylation of blood and thus to correct hypercapnia and respiratory acidosis [1, 2]. ECCO_2_R is similar to extracorporeal membrane oxygenation (ECMO) but uses lower blood flow, usually less than 1500 mL/min. Therefore, this technique has little or no impact on blood oxygenation. Initially, ECCO_2_R was developed in the treatment of patients with acute respiratory distress syndrome (ARDS) [3], but because of the progressive improvement of this technique and its use in hospitals, ECCO_2_R could be proposed as a therapeutic option in cases of hypercapnic respiratory insufficiency, either during acute and severe decompensation of chronic obstructive pulmonary disease (COPD) [4] or in ARDS to achieve less invasive mechanical ventilation (IMV) [5]. In this review of the literature, we will discuss the current knowledge on the pathophysiology related to hypercapnic respiratory failure, the principles of the ECCO_2_R technique, and its place in the treatment of ARDS and acute and severe decompensations of COPD.

## ECCO_2_R: from applied physiology to clinical studies

### ***Pathophysiological rationale of the use of ECCO***_***2***_***R in COPD exacerbations***

The amount of CO_2_ in the blood is higher than that of oxygen. CO_2_ is mainly present in blood as bicarbonates and to a lesser extent in dissolved form, whereas O_2_ is mainly linked to hemoglobin. Small variations in the partial pressure of CO_2_ (PaCO_2_) cause significant variations in the level of CO_2_ in the blood, unlike the relationship between the O_2_ partial pressure and O_2_ blood content. Therefore, extracorporeal CO_2_ removal can be realized with lower blood flow rates than requires extracorporeal oxygenation but with enough fresh gas flow sweeping the exchange membrane [6].

ECCO_2_R aims to eliminate blood CO_2_ to fight against potential adverse effects of hypercapnia and related acidosis. Hypercapnia has deleterious extrapulmonary consequences, particularly on the brain, by increasing cerebral blood flow and therefore intracranial pressure [7]. In addition, in the lungs, hypercapnia leads to pulmonary vasoconstriction, increases right ventricular afterload, and decreases myocardial contractility with consequent right heart failure [8]. Moreover, hypercapnic acidosis may further damage the lungs by increasing both nitric oxide production and inflammation and altering alveolar epithelial cells [9]. Finally, because of its immunosuppressive properties, hypercapnic acidosis may exacerbate lung damage by exacerbating pulmonary bacterial infections [9].

During exacerbations of COPD, the volume of CO_2_ removed by the lungs is reduced due to worsening dynamic overdistension and the gap between ventilation and perfusion [10], accompanied by severe hypercapnia. In addition, in patients with COPD exacerbation, CO_2_ production is estimated to be 23% higher than the normal value of 200 to 250 mL/min due to increased respiratory muscle work and metabolism [10].

**Fig. 1:**
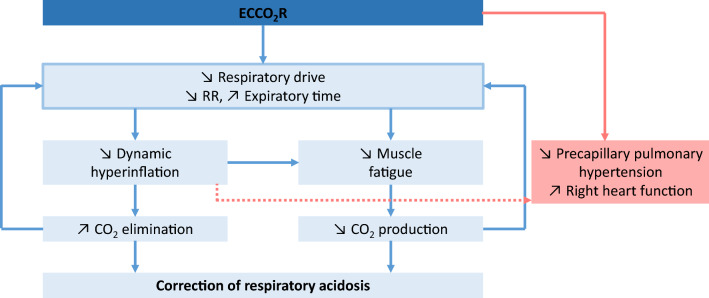
Pathophysiological rationale for the use of ECCO_2_R in COPD exacerbations

Therefore, during an exacerbation of COPD, relieving the native lungs of at least a portion of the CO_2_ could potentially improve the acid–base balance and reduce the patient's respiratory work, resulting in a reduced respiratory rate and alveolar ventilation [11]. Instead of mechanically increasing alveolar ventilation with IMV in an already hyperinflated lung to increase CO_2_ removal, the use of ECCO_2_R may allow a decrease in respiratory volume and respiratory rate, resulting in longer expiratory time that is better adapted to the high expiratory time constant of the respiratory system. Through these physiological mechanisms, ECCO_2_R can neutralize the vicious cycle of dynamic hyperinflation and its harmful respiratory and cardiovascular consequences. Beneficial effects derived from respiratory mechanics, ventilatory muscle efficiency, respiration, and cardiovascular function can improve gas exchange and relieve dyspnea, potentially preventing the failure of NIV or facilitating weaning from IMV [10–12]. The pathophysiological rationale for the use of ECCO_2_R in COPD exacerbation is presented in Fig. 1 (Pathophysiology of respiratory acidosis is presented in Additional file 1 and pathophysiology of COPD is presented in Additional files 1 and 2 (Figure S1)).

### ***Pathophysiological rationale of the use of ECCO***_***2***_***R in ARDS***

In recent decades, very important progress has been made in the understanding of the pathophysiology of ARDS. The recognition of ventilatory-induced lung injury (VILI) has led to drastic changes in the ventilatory management of these patients [13, 14]. The historical trial conducted by the ARDSNet group demonstrated that the ventilation of ARDS patients with a low tidal volume (VT) of 6 mL/kg (vs. 12 mL/kg) significantly reduced mortality [15]. However, recent results have shown that pulmonary hyperinflation still occurs in approximately 30% of ARDS patients despite this so-called “protective” ventilation [16]. This analysis suggests a beneficial effect of VT reduction, even in patients already at a plateau pressure (Pplat) < 30 cm H_2_O [17]. The decrease in the VT and Pplat will also decrease the driving pressure, which has recently been identified as a major risk factor for mortality in ARDS patients [18]. A reduction in VT to less than 6 mL/kg to reach a low Pplat level may induce severe hypercapnia that may increase intracranial pressure, causes pulmonary hypertension, decreases myocardial contractility, reduce renal blood flow, and releases endogenous catecholamines [19, 20]. In a recent multicenter study on 35 ARDS patients with PaO_2_/FiO_2_ ≤ 150 mmHg, Richard et al. reduced VT to 4 mL/kg and further adjusted respiratory rate (RR) to keep pH ≥ 7.20. RR was augmented up to 40 breaths/min. On day 2, VT decreased from 6.0 [5.9–6.1] to 4.1 [4.0–4.7] ml/kg leading to a significant decrease in driving pressure from 12 [9–15] to 8 [6–11] cmH_2_O. They concluded that ultra-low tidal volume ventilation may be applied in approximately 2/3 of moderately severe-to-severe ARDS patients while 2 patients (6%) developed acute cor pulmonale and 11 patients (32%) developed transient severe acidosis with pH < 7.15. A 4 cmH_2_O median reduction in driving pressure has been reached, at the price of transient episodes of severe acidosis [21]. This strategy is therefore not feasible for most ARDS patients with conventional IMV [22]. Therefore, ECCO_2_R could be used to achieve a VT < 6 mL/kg, thus lowering the Pplat, driving pressure and mechanical power [23–27] while maintaining PaCO_2_ and pH in physiological standards.

## Technical principles

**Fig. 2:**
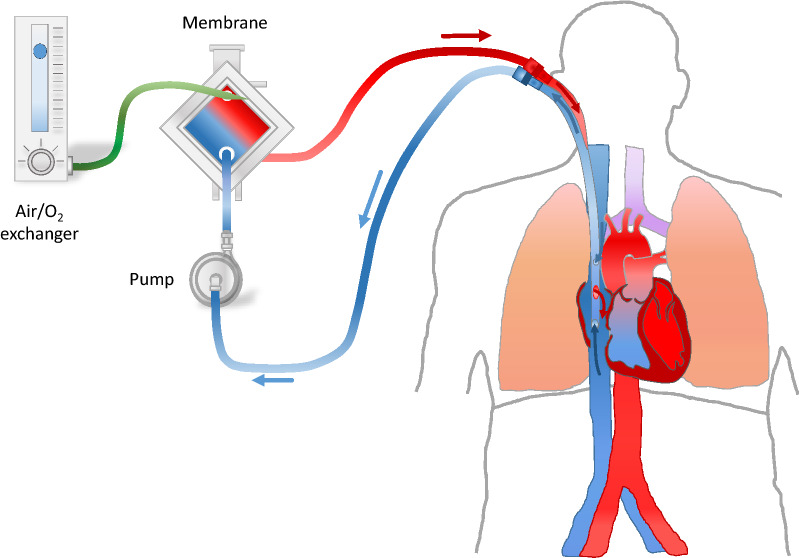
Venovenous ECCO_2_R system with pump. ECCO_2_R: extracorporeal carbon dioxide removal

**Fig. 3:**
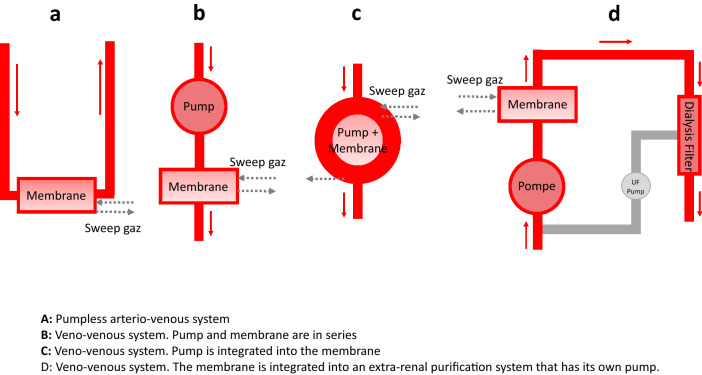
Schematic representation of different ECCO_2_R systems. ECCO_2_R: extracorporeal carbon dioxide removal. **a** Pumpless arteriovenous system. **b** Venovenous system. Pump and membrane are in series. **c** Venovenous system. Pump is integrated into the membrane. **d** Venovenous system. The membrane is integrated into an extrarenal purification system that has its own pump

**Fig. 4:**
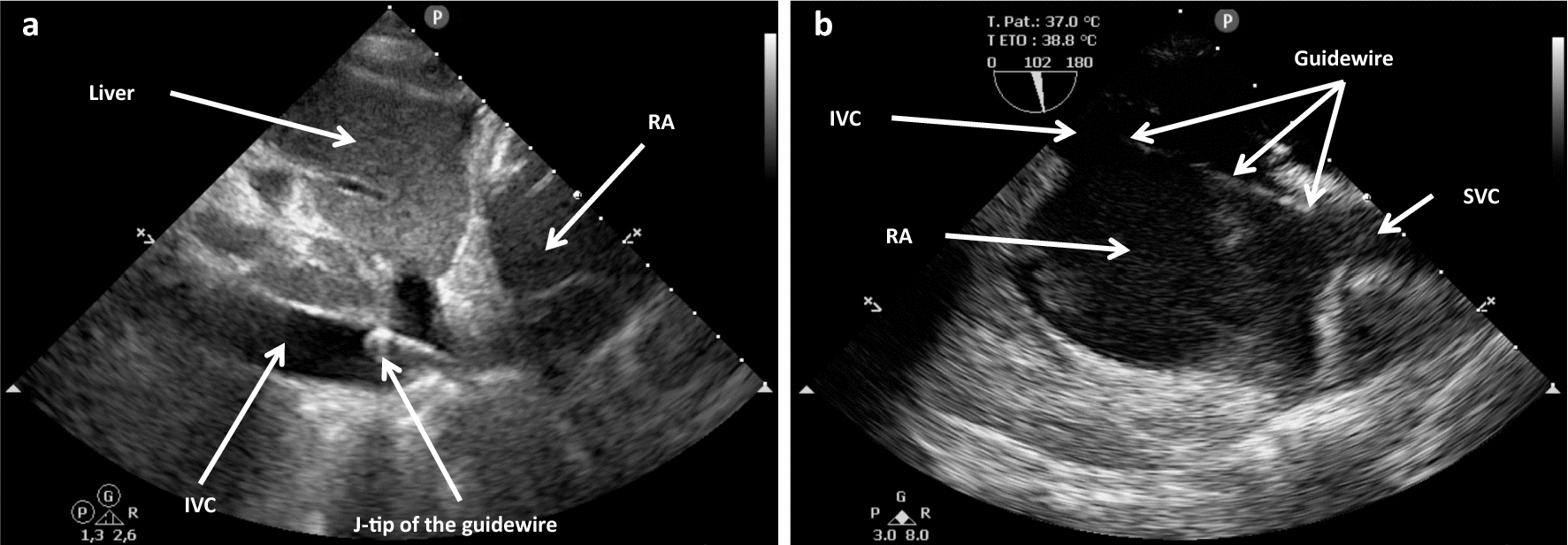
**a** Transthoracic echocardiography subcostal view showing the J-tip of the guidewire entering the inferior vena cava. **b** Transesophageal echocardiography bicaval view showing the guidewire passing through from the superior vena cava into the right atrium and entering into the inferior vena cava. *RA* right atrium, *SVC* superior vena cava, *IVC* inferior vena cava

Catheters or cannulas are needed to implement this technique. There are two categories of ECCO_2_R. The first category is the so-called arteriovenous technique, where the removal of CO_2_ is possible without a pump. A femoro-femoral approach is used. This technique requires arterial and venous cannulation with 15 French cannulas. The blood flow inside the device depends exclusively on the cardiac output of the patient, which explains the great variability of the ability to oxygenate the patient. However, with a membrane surface of 1.3 m^2^, its decarboxylation capacities are satisfactory. The second technique is called the venovenous technique, where the use of a pump is necessary (Fig. 2). The venovenous technique uses low or very low blood flow. Currently, it is the venovenous technique that is conventionally used for ECCO_2_R. The pumps used are rollers, centrifugal or diagonal, electric or electromagnetic. Figure 3 shows a schematic representation of different ECCO_2_R systems. The gas exchange membrane is a device with a complex geometry based on hollow fibers. The material used is poly-4-methyl-1-pentene (PMP). The exchange surfaces vary in size from 0.32 to 0.65 m^2^ for venovenous systems and 1.3 m^2^ for arteriovenous systems. Circuits such as membranes are coated with heparin for better biocompatibility, better gas exchange and less capillary leakage. The extraction of carbon dioxide is done through the sweeping of the membrane by a fresh gas (O_2_ or medical air) devoid of CO_2_. Current systems used to remove CO_2_ are venovenous and use double-lumen venous catheters/cannulas. The venous approach is classically achieved through the right internal jugular or femoral vein, and puncture of the vessel is performed under ultrasound guidance. The placement of the guidewire and the cannula requires control by transesophageal or subxiphoid transthoracic echocardiography (Fig. 4). Anticoagulation therapy (anti-Xa activity between 0.3 and 0.6 IU/L) is mandatory to avoid thrombosis in the circuit. Thus, any patient with a contraindication to anticoagulation therapy cannot benefit from ECCO_2_R. There are different types of machines on the market. The devices adapted from the VV-ECMO technique are very effective for CO_2_ removal but require the insertion of cannulas between 18 and 19 French. The blood flow generated is between 500 and 1500 mL/min. The newest ECCO_2_R devices are relatively simple to use because they require the insertion of a smaller double-lumen cannula (up to 13–15 Fr) and work with very low blood flow rates (between 0.2 to 0.5 L/min). However, their CO_2_ removal performance remains limited [11]. The characteristics of the different ECCO_2_R systems available on the market are summarized in Table 1.

**Table 1:** Characteristics of the different ECCO_2_R and VV-ECMO systems

	Partial extracorporeal support (ECCO_2_R)				Total extracorporeal support (ECMO)	
	Very low flow	Low flow	Intermediate flow	Intermediate flow	High flow	High flow
Blood flow (L/min)	200–400	400–500	500–1000	500–4500	2500–5000	2500–7000
Vascular access	Venovenous	Venovenous	Venovenous	Arteriovenous	Venovenous	Venovenous
Cannula size	13 Fr	15.5 Fr	18–19 Fr	15 Fr	27–31 Fr	Drainage: 25–29 Fr
Cannula configuration	Dialysis catheter	Double-lumen cannula	Double-lumen cannula	Arterial and venous cannulae	Double-lumen cannula	Reinjection: 17–21 Fr
Priming volume (mL)	140–160	200–300	250–350	175	300–500	300–500
Anti-Xa activity (UI/L)	0.3–0.4	0.3–0.4	0.3–0.4	0.3–0.4	0.2–0.3	0.2–0.3
Membrane surface (m^2^)	0.32	0.59	0.65	1.3	1.8	1.8
CO_2_ extraction (% of initial value)	< 25	25	50	50–60	> 50	> 50
O_2_ transfer (mL/min)	Ø	10	20	20–50	150–300	150–350

*ECCO*_*2*_*R* extracorporeal carbon dioxide removal, *VV-ECMO* venovenous extracorporeal membrane oxygenation

## Use of ECCO_2_R in severe acute exacerbations of COPD

Noninvasive ventilation remains the gold standard for the treatment of acute hypercapnic respiratory failure [28], but in approximately 20 to 30% of cases, this technique may not be sufficient, and patients need to be intubated and mechanically ventilated. The mortality of patients requiring the use of IMV is higher than those receiving NIV alone. Thus, the combination of ECCO_2_R therapy with NIV could be a therapeutic option to reduce the failure of NIV and prevent the use of intubation and IMV. In fact, the use of ECCO_2_R in patients with hypercapnic respiratory failure may improve the efficacy of NIV in terms, that ECCO_2_R, decreases respiratory rate, and reduces dynamic hyperinflation and intrinsic PEEP. In addition, by avoiding the use of IMV and therefore endotracheal intubation, it is also possible to limit the adverse effects related to analgo-sedation, which include hemodynamic instability, difficult and prolonged respiratory weaning, and a significant number of neurological disorders related to prolonged sedation. The absence of analgo-sedation also allows patients to drink, eat, communicate with relatives, and perform active physiotherapy. In addition, it has recently been demonstrated that ECCO_2_R, by decreasing the respiratory rate, can reduce the work of breathing and decrease the CO_2_ production of the respiratory muscles. Therefore, this contributes to the decrease in PaCO_2_ [29]. As a result, this may facilitate weaning from IMV and promote earlier extubation.

## Use of ECCO_2_R decreases the use of IMV in patients with COPD exacerbation

Kluge et al. [30] investigated the feasibility of a pumpless extracorporeal assist (PECLA) system in 21 patients with COPD who did not respond to NIV. The use of PECLA system was associated with decreased PaCO_2_ levels and improved pH after 24 h and avoided the use of intubation and IMV in 90% of treated patients. Retrospective analysis with a control group showed no significant difference in mortality at 28 days (19% with ECCO_2_R and 24% without ECCO_2_R) or at 6 months (both groups at 33%) or in the median duration of ICU or hospital length of stay (15 vs 30 days and 23 vs 42 days, respectively). In the study conducted by Burki et al. [4], 20 hypercapnic patients with COPD were treated with ECCO_2_R using a 15.5-Fr dual-lumen cannula, allowing an average blood flow of 430 mL/min. The authors reported improvement in both hypercapnia and respiratory acidosis, and IMV was avoided in the nine patients treated with NIV. More recently, Del Sorbo et al. [31] reported that ECCO_2_R with a 14-Fr dual-lumen catheter and blood flow rates of 177 to 333 mL/min not only improved respiratory acidosis, but also reduced the need for intubation in 25 patients with COPD who have a high risk of NIV failure. Compared with the control group, intubation risk and hospital mortality were significantly lower in the ECCO_2_R group. These results were challenged in a recent study by Braune et al. [32], which showed that IMV was avoided in 56% of patients treated with ECCO_2_R but was associated with a higher incidence of complications. However, several differences were found between these two studies, including the inclusion of patients with contraindications for NIV and the unexpectedly high incidence of hypoxemic patients [33]. In another study, Morelli et al. [34] confirmed the efficacy of ECCO_2_R (with a flow rate of 250 to 450 mL/min via a 13-Fr dual-lumen catheter) to reduce PaCO_2_ in a case series of 30 patients with acute hypercapnic respiratory failure due to COPD exacerbation who refused endotracheal intubation after NIV failure. The duration of ECCO_2_R treatment was 2 to 16 days, and it was possible to avoid endotracheal intubation in 27 patients. Finally, in a round table, 14 European experts' views were collated to better understand how ECCO_2_R therapy is used, how patients are selected and managed. In COPD patients with acute exacerbation, a consensus was found that, in patients at risk of NIV failure, no decrease in PaCO_2_ and no decrease in respiratory rate were principal criteria for starting with ECCO_2_R therapy. Main treatment targets in COPD patients were patient well-being, pH (> 7.30–7.35), respiratory rate (< 20–25 breaths/min), decrease of PaCO_2_ (by 10–20%), weaning from NIV, decrease in HCO_3_^−^ and maintaining hemodynamic stability [35].

## Use of ECCO_2_R to help weaning from IMV

In the case series of Elliot et al. of two patients with severe acute asthma [36], the addition of pumpless ECCO_2_R to IMV corrected hypercapnia and related acidosis and reduced other support measures, including hemodynamics, and allowed weaning from IMV. In the study by Burki et al. [4], in the subgroup of 11 mechanically ventilated patients, ECCO_2_R allowed weaning from IMV in only 3 patients. Nevertheless, even if they were not completely weaned, in three other patients, ventilatory assistance could be reduced. Using a double-lumen cannula (20–23 Fr) with a blood flow of 1 to 1.7 L/min, Abrams et al. [37] successfully weaned and extubated five COPD patients with acute respiratory acidosis after only 24 h of IMV. All of these patients survived until discharge from the hospital. Similarly, using a pediatric VV-ECMO system (with blood flow rates of 0.9 L/min through a 19-Fr double-lumen cannula placed in the right internal jugular vein) in two adult patients with COPD exacerbation, Roncon-Albuquerque Jr [38] reported early extubation after 72 h and patient mobilization on day 6. A retrospective analysis of data from 12 patients with hypercapnic respiratory failure confirms the efficacy of ECCO_2_R with median blood flow rates of 1.2 to 1.4 L/min in the correction of hypercapnia and in the reduction of ventilation pressures and minute ventilation. Of the patients studied, six patients with mainly hypercapnic pulmonary insufficiency due to COPD or fibrosis were promptly weaned off of IMV and survived until discharge from the hospital. It should be noted that five patients were awake and spontaneously breathing during ECCO_2_R therapy [39].

Taken together, these results support the notion that ECCO_2_R may be useful for the avoidance of intubation during NIV and for the facilitation of weaning from IMV. Nevertheless, the observational nature of the available data makes it difficult to understand the efficacy and safety of such strategies in these patients.

The relevant clinical studies on ECCO_2_R in COPD are summarized in Table 2.

**Table 2:** Relevant clinical studies on ECCO_2_R in COPD

Studies	Type	Number of patients	Characteristics of ECCO_2_R				Duration of ECCO_2_R	Main results
			Configuration	Blood flow (mL/min)	Fresh gas flow (L/min)	Membrane surface (m^2^)		
ECCO_2_R with mixed indications								
Burki et al.	Pilot study	20	VV configuration via a 15.5-Fr double-lumen catheter (femoral or jugular)	430	Not reported	PLP based on siloxane layer; 0.59 (ALung Hemolung RAS)	2–192 h	An ECCO2R was implanted in 20 patients with hypercapnic COPD in three distinct groups: group 1 (*n* = 7) patients under NIV with a high risk of MV; group 2 (*n* = 2) patients who could not be weaned from NIV; and group 3 (*n* = 11) patients under MV without possible weaning. MV was avoided in all patients in group 1 Both patients in group 2 were weaned off of NIV In group 3, three patients were weaned from MV and MV could be reduced in two patients. One patient died from retroperitoneal hemorrhage (during cannulation)
ECCO_2_R to avoid mechanical ventilation								
Del Sorbo et al.	Paired cohort study with historical control	25	Continuous VV hemofiltration system modified by a pulmonary membrane via a 14-Fr double-femoral cannula (femoral)	255	8	PLP; 1.35 (Hemodec DecapSmart®)	1–2 h	Significantly higher risk of intubation in the NIV-only group (HR 0.27, 95% CI 0.07–0.98). Thirteen patients experienced adverse events: 3 bleeding, 1 venous perforation and 9 device malfunctions
Braune et al.	Case–control study	25	VV configuration via a single double-lumen cannula of 22 or 24 Fr (femoral or jugular)	1300	Not reported	PMP; 1.3 (Novalung iLA activve)	8.5 h	Intubation was avoided in 14 of 25 patients under ECCO2R (56%). Seven patients under ECCO2R were intubated due to progressive hypoxemia and four due to ventilatory failure despite ECCO2R and NIV. Nine patients under ECCO2R (36%) had major bleeding complications. The 90-day mortality was 28% vs 28%
Morelli et al.	Retrospective study	30	VV configuration via a 13-Fr catheter (femoral or jugular)	250–450	Not reported	Not reported	2–16 h	Thirty patients with acute hypercapnic respiratory failure due to exacerbation of COPD who refused endotracheal intubation after the failure of NIV It was possible to avoid endotracheal intubation in 27 patients
Kluge et al.	Retrospective study	21	Arteriovenous femoral with arterial cannula of 13 to 15 Fr and venous cannula from 13 to 17 Fr	1100	Not reported	PMP; 1.3 (iLA®)	9 h	Nineteen (90%) patients in the PECLA group did not need intubation No significant difference in 28-day mortality (24 vs. 19%, *p* = 0.85), 6-month mortality (33 vs. 33%), or length of hospital stay (23 vs 42 days, *p* = 0.06) Much less tracheostomy in the PECLA group (10 vs. 67%, *p* = 0.004) Two major bleeds and seven minor bleeds in the PECLA group
ECCO_2_R to wean from mechanical ventilation								
Abrams et al.	Pilot prospective study	5	VV configuration via a 20–24 Fr double-lumen jugular catheter using a lower flow rate on the ECMO system	1700	1–7	PMP; 0.98 (Maquet PALP CardioHelp)	8 h	The mean time (± SD) until ambulation after the start of ECCO2R was 29.4 ± 12.6 h Four patients were sent home and one received a lung transplant. Only two minor bleeding complications
Roncon-Albuquerque Jr et al.	Case series	2	VV configuration via a dual-lumen 19-Fr cannula (Avalon Elite®)	700–1000	2–12	PMP, 0.8 (Maquest Quadrox –i pediatric)	7–8 h	ECCO2R was effective in exacerbations of COPD requiring MV, allowing the correction of acute respiratory acidosis, early extubation after 72 h, and early mobilization at day 6. No device-related complications were observed
Hermann et al.	Retrospective study	12	VV configuration via a single double-lumen 22 or 24-Fr cannula (femoral or jugular)	900–2100	2–12	PMP; 1.3 (Novalung iLA activve)	2–30 h	The indication for ECCO_2_R was hypercapnia due to terminal pulmonary insufficiency awaiting lung transplantation, pneumonia and COPD or severe acute asthma ECCO_2_R allowed efficient decarboxylation, leading to a reduction in ventilation pressures and facilitating spontaneous respiration. Five patients were weaned from VM and extubated under ECCO_2_R
Elliot et al.	Case series	2	Arteriovenous configuration without femoro-femoral pump	1400–1600	10–15	PMP; 1.3 (iLA®)	4 h	A 74-year-old man and a 52-year-old woman with severe life-threatening asthma who developed progressive hypercapnia and severe acidosis refractory to all other treatments. The addition of a pumpless ECCO_2_R to the MV corrected hypercapnia and secondary acidosis and reduced other support measures, including hemodynamics and weaning from MV

*ECCO*_*2*_*R* extracorporeal carbon dioxide removal, *PMP* poly-4-methyl-1-pentene, *PLP* polypropylene, *PECLA* pumpless extracorporeal lung assist

## Use of ECCO_2_R in acute respiratory distress syndrome (ARDS)

The latest feasibility and safety pilot study of 20 patients with moderate and/or severe ARDS, in whom ECCO_2_R was performed with a new standalone platform (without concomitant extrarenal treatment), Prismalung® (Gambro-Baxter), integrated on the Prismaflex® platform (Gambro-Baxter), showed a reduction in the tidal volume from 6 to 4 mL/kg of the predicted body weight and in Pplat below 25 cmH_2_O, thus achieving ultraprotective ventilation. However, the results show that despite maximal ECCO_2_R treatment (sweep gas flow at 10 ± 0.3 L/min and blood flow at 421 ± 40 mL/min, corresponding to the maximum that this platform can generate), patients ventilated at 4 mL/kg of their predicted body weight become acidotic (pH decreased from 7.39 ± 0.1 to 7.32 ± 0.10 and PaCO_2_ increased from 43 ± 8 mmHg to 53 ± 9 mmHg) [5].

A larger prospective multicenter international phase II study aimed to assess the feasibility and safety of extracorporeal carbon dioxide removal (ECCO_2_R) to facilitate ultraprotective ventilation (V_T_ 4 mL/kg and Pplat ≤ 25 cmH_2_O) in patients with moderate ARDS. The primary endpoint was the proportion of patients achieving ultraprotective ventilation with PaCO_2_ not increasing more than 20% from baseline and arterial pH > 7.30. Both lower CO_2_ extraction and higher CO_2_ extraction devices (membrane lung cross-sectional area 0.59 vs. 1.30 m^2^; flow 300–500 mL/min vs. 800–1000 mL/min, respectively) were used in this study. 59 patients were included. The proportion of patients who achieved ultraprotective settings by 8 h and 24 h was 78% (74 out of 95 patients; 95% confidence interval 68–89%) and 82% (78 out of 95 patients; 95% confidence interval 76–88%), respectively. ECCO_2_R was maintained for 5 [3–8] days. A total of 69 patients (73%) were alive at day 28. Fifty-nine patients (62%) were alive at hospital discharge. The authors concluded that the use of ECCO_2_R to facilitate ultraprotective ventilation was feasible [40]. In the recent round table of European experts on ECCO_2_R, an agreement was reached that the main treatment goal of ECCO_2_R therapy in patients with ARDS was to carry out ultraprotective lung ventilation through handling CO_2_ levels. Driving pressure with plateau pressure optimization was estimated as the principal criteria for ECCO_2_R introduction. Main targets for patients with ARDS starting with ECCO_2_R included pH (> 7.30), respiratory rate (< 25 or < 20 cycles/min), P_plat_ (< 25 cmH_2_O) and driving pressure (< 14 cmH_2_O) [35]. Finally, using data from the SUPERNOVA trial (95 patients with early moderate ARDS), Goligher et al. assessed the independent effects of alveolar dead space fraction (ADF), respiratory system compliance (Crs), hypoxemia (PaO_2_/FiO_2_), and device performance (higher vs lower CO_2_ extraction) on the magnitude of reduction in *V*_t_, driving pressure and mechanical power permitted by ECCO_2_R were assessed. The authors demonstrated that patients with higher ADF or lower Crs and patients treated with higher CO_2_ extraction are most likely to benefit from ECCO_2_R [41].

The combination of continuous renal replacement therapy (CRRT) and ECCO_2_R with very low blood flow is a promising concept. The hypothesis in a study by Moerer et al. is that this combined system can remove enough CO_2_ to facilitate protective ventilation in mechanically ventilated patients. In 11 ventilated patients with acute renal failure placed under CRRT, a very-low-flow ECCO_2_R (300 mL/min) was added to the circuit. During 6 h of combined therapy, the elimination of CO_2_ and its effect on the possibility of achieving protective ventilation were evaluated. The ventilation settings were maintained in assisted mode or in controlled pressure mode, allowing spontaneous breathing. With very-low-flow ECCO_2_R, a significant decrease in minute ventilation, tidal volume and paCO_2_ was possible after 1–3 h but not after 6 h of treatment. On the other hand, no significant reduction in the driving pressure was observed during the combined treatment. The CO_2_ removal was 20.73 mL CO_2_/min. Therefore, the very low blood flow in ECCO_2_R associated with CRRT treatment is not enough to significantly reduce respiratory work. The absolute cause could be the absolute amount of CO_2_ removed by approximately 10% of CO_2_ production in the resting adult. Therefore, the effectiveness of ECCO_2_R with very low blood flow in allowing protective ventilation is very limited [42]. Moreover, in another recent study including 20 hypercapnic ARDS patients requiring CRRT who were treated with a system combining very-low-flow ECCO_2_R (membrane lung 0.32 m^2^) and renal replacement therapy, the pH increased from 7.18 ± 0.09 to 7.22 ± 0.08 (*p* < 0.05). There was a significant reduction in ventilation requirements with a decrease in tidal volume from 6.2 ± 0.9 to 5.4 ± 1.1 mL/kg PBW (*p* < 0.05), associated to a reduced pulmonary stress and strain [43]. Even if these results were statistically significant, we can question their clinical relevance. The relevant clinical studies on ECCO_2_R in ARDS are summarized in Table 3.

**Table 3:** Relevant clinical studies on ECCO_2_R in ARDS

Studies	Type	Number of patients	Characteristics of ECCO_2_R				Duration of ECCO_2_R	Main results
			Configuration	Blood flow (mL/min)	Fresh gas flow (L/min)	Membrane surface (m^2^)		
ECCO_2_R in ARDS								
Augy et al. 31452899	Multicenter, observational, prospective, cohort study	70	VV configuration via a double-lumen 15.5-Fr venovenous catheter (either right jugular or femoral site) or a double-lumen 18 Fr (right jugular site) or 24 Fr (femoral site) or Novaport Twin (18, 22, or 24 Fr) catheters	430	Not reported	PLP based on siloxane layer; 0.59 (ALung Hemolung RAS) or PMP; 1.3 (Novalung iLA activve)	5 days	Main indications were ultraprotective ventilation for ARDS patients (*n* = 24), shortening the duration of IMV in COPD patients (*n* = 21), preventing intubation in COPD patients (*n* = 9), and controlling hypercapnia and dynamic hyperinflation in mechanically ventilated patients with severe acute asthma (*n* = 6). A reduction in median *V*_T_ was observed in ARDS patients, from 5.9 to 4.1 ml/kg (*p* < 0.001). A reduction in PaCO_2_ values was observed in AE-COPD patients, from 67.5 to 51 mmHg (*p* < 0.001). Median duration of ECCO_2_R was 5 days (IQR 3–8). Reasons for ECCO_2_R discontinuation were improvement (*n* = 33), ECCO_2_R-related complications (*n* = 18), the limitation of life-sustaining therapies or decision measures (*n* = 10), and death (*n* = 9). Main adverse events were hemolysis (*n* = 21), bleeding (*n* = 17), and lung membrane clotting (*n* = 11), with different profiles between the devices. Thirty-five deaths occurred during the ICU stay, 3 of which were ECCO_2_R related
Grasselli et al. 31425258	Retrospective study	11	VV configuration via a single double-lumen catheter 13 Fr (femoral or jugular)	333	Not reported	PMP; 1.8 (ProLUNG® ESTOR)	7 days	Twenty-four hours of ECCO_2_R treatment reduced arterial PaCO_2_ from 63 ± 12 to 54 ± 11 mmHg (*p* < 0.01), increased arterial pH from 7.29 ± 0.07 to 7.39 ± 0.06 (*p* < 0.01), and decreased respiratory rate from 32 ± 10 to 21 ± 8 bpm (*p* < 0.05). All four ARDS patients were invasively ventilated at the initiation of treatment, no one was extubated and they all died. Among the seven patients with an exacerbation of COPD, four were managed with noninvasive ventilation via ECCO_2_R, while three were extubated after starting ECCO2R. None of these seven patients was intubated or reintubated after ECCO2R and five (71%) survived to hospital discharge
Karagiannidis et al. 31014366	Physiological study	20	VV configuration via a 19 Fr/38 cm femoral-draining cannula and a 17 Fr/15 cm inlet-flow cannula (Maquet, Rastatt, Germany)	2000–3000	0–10	PMP; 1.3 (Maquet HLS CardioHelp)	Not reported	Patients supported by NIV-NAVA were studied during stepwise weaning of ECCO_2_R. Based on dyspnea, tolerance, and blood gases, weaning from ECCO_2_R was successful in 12 patients and failed in 8 patients. Respiratory drive increased to 19 ± 10 μV and 56 ± 20 μV in the successful and unsuccessful weaning groups, respectively, resulting in all patients keeping their CO_2_ and pH values stable. Eventually, 19 patients were discharged, while one patient died. Mortality at 90 days and 180 days was 15% and 25%, respectively
Schmidt et al. 29743094	Prospective study	20	A 13-Fr hemodialysis venous catheter (Gamcath™®; Gambro-Baxter)	420	10	PMP, 0.32 (PrismaLung®; Gambro-Baxter)	31 h	Twenty patients with mild (*n* = 8) or moderate (*n* = 12) ARDS were included. VT was gradually lowered from 6 to 5, 4.5, and 4 ml/kg, and PEEP was adjusted to reach 23 ≤ *P*_plat_ ≤ 25 cmH_2_O. While VT was reduced from 6 to 4 ml/kg and *P*_plat_ was maintained at < 25 cmH_2_O, PEEP was significantly increased from 13.4 ± 3.6 cmH_2_O at baseline to 15.0 ± 3.4 cmH_2_O, and the driving pressure was significantly reduced from 13.0 ± 4.8 to 7.9 ± 3.2 cmH_2_O (both *p* < 0.05). The PaO_2_/FiO_2_ ratio and respiratory system compliance were not modified. Mild respiratory acidosis occurred, with mean PaCO_2_ increasing from 43 ± 8 to 53 ± 9 mmHg and mean pH decreasing from 7.39 ± 0.1 to 7.32 ± 0.10 from baseline to 4 ml/kg VT, respectively. Day 28 mortality was 15%
Peperstraete et al. 29179681	Prospective pilot study	10	A 13.5-Fr double-lumen catheter (Niagara**™**, Bard)	300–500	0–7	PMP; 0.67 (Lilliput 2, LivaNova)	5 days	ARDS patients on MV, with PaO_2_/FiO_2_ < 150 mmHg, tidal volume ≤ 8 mL/kg with positive end-expiratory pressure ≥ 5 cmH_2_O, FiO_2_ titrated to SaO_2_ 88–95%, plateau pressure ≥ 28 cmH_2_O, and respiratory acidosis (pH < 7.25). After 2 h of ECCO_2_R, 6 patients had a ≥ 20% decrease in PaCO_2_ (60%); PaCO_2_ decreased 28.4% (from 58.4 to 48.7 mmHg, *p* = 0.005), and pH increased (1.59%, *p* = 0.005). 6 patients had an AE (60%); bleeding occurred in 5 patients (50%) and circuit thrombosis in occurred in 3 patients (30%). These adverse events were judged not to be life threatening
Hilty et al 28638160	Retrospective study	20	VV configuration via a single double-lumen 13-Fr catheter (femoral or jugular)	300–350	Not reported	PMP; 1.8 (ProLUNG® ESTOR)	1.4–5.2 days	Causes of HRF were severe ARDS (*N* = 11), COPD (*N* = 4), chronic lung transplant rejection (N = 3) and cystic fibrosis (*N* = 2). During the first 8 h of ECCO_2_R, PaCO_2_ decreased from 10.6 (9.3–12.9) to 7.9 (7.3–9.3) kPa (*p* < 0.001) and pH increased from 7.23 (7.09–7.40) to 7.36 (7.27–7.41) (*p* < 0.05). Lung protective tidal volume (4.7 (3.8–6.5) mL/kg) and peak ventilator pressure (28 (27–30) mbar at 24 h) were maintained. Thrombocyte count decreased by 52% (*p* < 0.01) and fibrinogen decreased by 38% *(p* < 0.05). Intubation could be avoided in all spontaneously breathing patients. In 4/6 patients, high blood flow extracorporeal circulation was required due to increased oxygen demand. Six of 14 mechanically ventilated patients recovered from respiratory support
Moss et al. 27195746	Retrospective study	14	VV configuration via a double-lumen 15.5-Fr venovenous catheter (either right jugular or femoral site)	440	9.6	PLP based on siloxane layer; 0.59 (ALung Hemolung RAS)	5 days	A statistically significant improvement in pH (*p* = 0.012) was demonstrated. Ten patients were discharged from the intensive care unit (ICU) alive. Four complications related to ECCO2R were reported, none of which resulted in serious adverse outcomes
Fanelli et al. 26861596	Prospective pilot study	14	VV configuration via a double-lumen 15.5-Fr venovenous catheter (either right jugular or femoral site)	435	10	PLP based on siloxane layer; 0.59 (ALung Hemolung RAS)	2 h	During the 2-h run-in phase, V_T_ reduction from baseline (6.2 mL/kg PBW) to approximately 4 mL/kg PBW caused respiratory acidosis (pH < 7.25) in all fifteen patients. Driving pressure was significantly reduced during the first two days compared to baseline (from 13.9 to 11.6 cmH_2_O; *p* < 0.05) and there were no significant differences in the values of respiratory system compliance. Rescue therapies for life-threatening hypoxemia such as prone position and ECMO were necessary in four and two patients, respectively. Only two study-related adverse events were observed (intravascular hemolysis and femoral catheter kinking)
Combes et al. 30790030	Prospective multicenter international phase II study	95	VV configuration via a double-lumen venovenous catheter (either right jugular (57%) or femoral (43%) site) Catheter size was 15.5 Fr (Hemolung) and 18 Fr (iLA activve and Cardiohelp® HLS 5.0)	300–500 mL/min vs. 800–1000 mL/min	6–10	PLP based on siloxane layer (ALung Hemolung RAS, iLA activve, Novalung, Cardiohelp® HLS 5.0, Getinge)		The proportion of patients who achieved ultraprotective settings by 8 h and 24 h was 78% (74 out of 95 patients; 95% confidence interval 68–89%) and 82% (78 out of 95 patients; 95% confidence interval 76–88%), respectively. ECCO_2_R was maintained for 5 [3–8] days. Six SAEs were reported; two of them were attributed to ECCO_2_R (brain hemorrhage and pneumothorax). ECCO_2_R-related AEs were reported in 39% of the patients. A total of 69 patients (73%) were alive at day 28. Fifty-nine patients (62%) were alive at hospital discharge
Peperstraete et al. 29179681	Paired cohort study with historical control	25	Continuous VV hemofiltration system modified with pulmonary membrane via 14-Fr double-femoral cannula (femoral)	255	8	PLP; 1.35 (Hemodec DecapSmart®)	1–2 h	Significantly higher risk of intubation in the NIV-only group (HR 0.27, 95% CI 0.07–0.98). Thirteen patients experienced adverse events: 3 bleeding, 1 venous perforation and 9 device malfunctions

*ECCO*_*2*_*R* extracorporeal carbon dioxide removal, *PMP* poly-4-methyl-1-pentene, *PLP* polypropylene, *PECLA* pumpless extracorporeal lung assist

## Role of ECCO_2_R while awaiting lung transplantation

It is well known that patients who develop acute gas exchange impairment requiring IMV while awaiting lung transplantation are more likely to die than patients who do not require IMV [44]. The reason for using ECCO_2_R in such patients is the possibility of the avoidance of endotracheal intubation and IMV, thus limiting their adverse effects (i.e., ventilator-associated pneumonia) that may preclude transplantation. In addition, by using ECCO_2_R, it is possible to avoid analgo-sedation, which allows the patient to maintain the tone of the respiratory muscles and to continue to perform active physiotherapy. Despite this pathophysiological rationale, studies regarding the use of ECCO_2_R in this subgroup of hypercapnic patients are still rare. Schellongowski et al. [45] performed a retrospective study of 20 patients with bronchiolitis obliterans, cystic fibrosis and idiopathic pulmonary fibrosis with indication for lung transplantation (*n* = 13) or retransplantation (*n* = 7). The use of venovenous ECCO_2_R and pumpless arteriovenous ECCO_2_R was associated with an improvement in hypercapnia and acidosis during the first 12 h of treatment. After a transition period of 4 to 11 days, 19 patients (95%) were successfully transplanted. Survival at the hospital was 75%. A very recent retrospective study confirmed that patients treated with ECCO_2_R before lung re-transplantation tended to have better survival [46]. In light of these findings, ECCO_2_R may even be useful in thoracic surgical procedures other than lung transplantation [47]. Nevertheless, given the complexity and the difficult clinical conditions of these patients awaiting lung transplantation, the use of ECCO_2_R in these patients should be performed only in experienced centers.

## ECCO_2_R-related complications and technical limitations

The use of ECCO_2_R may have pulmonary and hemodynamic consequences and may be associated with complications. Adverse events include events related to the patient, the circuit and mechanical events (Table 4). In four studies of ARDS patients, the use of ECCO_2_R was associated with hypoxemia and the need for an increase in FiO_2_. The present fact could be explained by lung derecruitment related to decrease in ventilation (favoring atelectasis). Moreover, PaO_2_/FiO_2_ worsening in ECCO_2_R may at least in part, reflect a modification of the alveolar gas content due to ECCO_2_R (modification of the respiratory quotient) [48]. To correct this phenomenon, IMV was implemented in spontaneously breathing patients [49] with both the use of high levels of PEEP and prone position to maintain functional residual capacity [24, 49–51]. In case of refractory hypoxemia a switch to VV-ECMO [52] was performed.

**Table 4:** Types of complications that can occur during treatment with ECCO_2_R

Types of complications	
Complications related to cannulation	Bleeding at vascular access
	Thrombosis
	Infection of the insertion site
	Accidental arterial insertion (venovenous system)
	Pneumothorax
	Hematoma
	Distal ischemia of the cannulated limb (arteriovenous system)
	Aneurysm (arteriovenous system)
	Pseudoaneurysm (arteriovenous systems)
Mechanical complications	Malfunction or failure of the pump
	Malfunction or failure of the membrane
	Malfunction or heater failure
	Thrombosis in the circuit/membrane
	Gas embolism
Complications related to patients	Aggravation of hypoxemia during the establishment of ultraprotective ventilation
	Bleeding in relation to anticoagulation
	Hemolysis
	Infection
	Heparin-induced thrombopenia

*ECCO*_*2*_*R* extracorporeal carbon dioxide removal

The major adverse effects may be caused by venous and/or arterial cannulation, with increased risk depending on the choice of vascular access and the type and size of cannulas. Transient ischemia of the lower limb, "false" aneurysm of the femoral artery [50] and fatal perforation following retroperitoneal bleeding have been described [4, 33].

Anticoagulation protocols with heparin are necessary to maintain the efficacy and performance of ECCO_2_R [53]. Thus, hemorrhagic events may be considered the most common complication and are associated with a higher number of blood transfusions during ECCO_2_R therapy [4, 30, 33, 49, 50, 52].

Transient thrombocytopenia, probably related to the use of heparin, has also been noted [4, 33, 51]. However, thrombocytopenia and decreased coagulation factors, certainly due to an activation of coagulation and fibrinolysis as well as an inflammatory response mediated by the complement system [54] may also be the result of interactions between blood components and the circuit. Future research should focus on improvements in anticoagulation protocols and the development of practical guidelines [55].

Despite anticoagulation protocols, clot formation in the circuits often occurs reducing the clearance of CO_2_ in the membrane and resulting in a rapid increase in PaCO_2_. The occurrence of membrane thrombosis should be considered a life-threatening event and necessitates rapid circuit changes, changes in ventilator parameters, and endotracheal intubation in the case of NIV [33, 51, 52]. Moreover, it seems that the reduction in blood flow through the membrane may be linked to an increase in the risk of thrombosis of the system. In the study of Schmidt et al. including 20 patients with mild or moderate ARDS, VT was gradually lowered from 6 to 5, 4.5, and 4 ml/kg. When arterial PaCO_2_ increased by > 20% from its initial value, a very-low-flow standalone ECCO_2_R was initiated to reduce respiratory acidosis. The authors showed that despite a heparin-infusion protocol that also included a bolus at catheter insertion, 50% of the treated patients experienced membrane clotting before the end of the experimental protocol [5]. In a retrospective study carried out by our group on 3 patients with severe COPD also assisted by a very-low-flow ECCO_2_R, thrombosis of the circuit occurred in 2 patients. In contrast, in our study, the 6 patients assisted by a higher blow flow ECCO_2_R did not experience circuit thrombosis [56]. It therefore appears that the blood flow passing throughout the circuit has a role in the occurrence of circuit thrombosis.

The displacement or twisting of the catheter/cannula may cause pump malfunction and promote thrombosis of the membrane. Finally, episodes of intravascular hemolysis have been reported in two case series, including one requiring a transfusion [51, 52].

Finally, CO_2_ extraction capacity differed between the devices available on the market. While re-analyzing the results of the SUPERNOVA trial according to the ECCO_2_R devices used (lower blood flow (area of membrane length 0.59 m2; blood flow 300–500 mL/min) vs higher blood flow (membrane area 1.30 m^2^; blood flow between 800 and 1000 mL/min), Combes et al. showed that reduction of V_T_ to 4 mL/kg was achieved in 55% and 64% of patients with the lower extraction versus 90% and 92% of patients with higher extraction devices at 8 and 24 h from baseline, respectively (*p* < 0.001) [57]. Moreover, ECCO_2_R-related hemolysis and bleeding were higher with lower than with higher extraction devices. In our retrospective study on COPD patients, we showed that when compared with a higher blood flow ECCO_2_R system, very low-flow device was not able to remove sufficient CO_2_, normalize pH or decrease respiratory rate [56].

## New technologies and ongoing research on ECCO_2_R

ECCO_2_R devices remove CO_2_ directly from the blood, facilitating ultraprotective ventilation or even offering an alternative to IMV. However, ECCO_2_R is not widely available, while dialysis is available in most intensive care units. Recent technological advances are focused on the development of minimally invasive devices that provide adequate CO_2_ removal with increased safety and simple use. Previous attempts to perform ECCO_2_R with dialysis by removing CO_2_ as bicarbonate have been affected by metabolic acidosis. Bicarbonate dialysis is possible, provided that the difference between the strong ions in the plasma is maintained. Using a mathematical model to study the effects of bicarbonate removal on pH and CO_2_ in plasma, in vitro experiments were performed to test CO_2_ removal using three dialysates with different bicarbonate concentrations (0, 16 and 32 mmol/L). This model predicts a reduction in partial CO_2_ pressure (PaCO_2_) and an increase in pH with a progressive reduction in plasma bicarbonate, provided that the strong ion difference and the maintenance of plasma proteins are preserved. In these in vitro experiments, CO_2_ removal with an adult-size filter was maximal with a dialysate not containing bicarbonate, equivalent to 94 mL/min (± 3.0) of CO_2_ eliminated. Under the same conditions, the dialysate containing a conventional concentration of bicarbonates (32 mmol/L) eliminated only 5 mL/min (± 4, *p* < 0.001). As expected, the pH increased after the removal of the bicarbonate. These data show that dialysis with low-bicarbonate dialysates is feasible and results in a reduction in plasma PaCO_2_. When scaled to estimate equivalent CO_2_ removal with an adult dialysis circuit, the amount eliminated competes with that of existing low-flow ECCO_2_R devices [58]. However, these methods may be impractical for clinical use due to acid–base disturbances, hemolysis, cardiac arrhythmias and micronutrient depletion despite several attempts to replace bicarbonate [59, 60]. Finally, other techniques were evaluated, including the combination of ECCO_2_R and continuous renal replacement therapy, the acidification of blood with lactic acid, the addition of carbonic anhydrase to the membrane and electrodialysis [60–62]. ECCO_2_R technique based on infusion of metabolizable acids exploits bicarbonate for gas exchange. An innovative lung support technique, called respiratory electrodialysis has been developed, consisting in a combination of a hemofilter, a membrane lung, and an electrodialysis unit. By applying electrodialysis to hemodiafiltrate, the pH and the electrolyte concentration are selectively modulated in specific sections of the extracorporeal circuitry. Blood is regionally acidified, bicarbonate is exchanged with chloride, and the PaCO_2_ is increased, leading to facilitated membrane lung CO_2_ removal [61]. These strategies can enhance the physiological benefits of ECCO_2_R while reducing its risks. However, studies demonstrating safety and efficacy are necessary before putting these technological innovations into clinical practice.

Several studies of ECCO_2_R are currently underway in patients with hypercapnic respiratory failure (ClinicalTrials.gov). Details of these studies are available in Additional file 2. These various ongoing clinical studies on the use of ECCO_2_R in COPD and ARDS are summarized in Additional file 2: Tables S1, S2, respectively.

## Conclusion

ECCO_2_R may be a promising adjunctive therapeutic strategy for the management of patients with severe COPD exacerbation and for the establishment of protective or ultraprotective ventilation in patients with ARDS without prognosis-threatening hypoxemia. To date, only the feasibility and the relative safety of this therapy have been studied and demonstrated and large randomized controlled studies are definitively warranted. In the meantime, a careful clinical evaluation of patients should be performed to select the most appropriate ECCO_2_R device in terms of extracorporeal blood flow and the potential complications of ECCO_2_R need to be considered.

## Take home messages

- Chronic obstructive pulmonary disease (COPD) exacerbation and protective mechanical ventilation of acute respiratory distress syndrome (ARDS) patients may induce hypercapnic respiratory acidosis.
- Extracorporeal carbon dioxide removal (ECCO_2_R) is an efficient technique which by eliminating blood CO_2_ fights against the adverse effects of hypercapnia and related acidosis.
- ECCO_2_R may be a promising adjunctive therapeutic strategy for the management of patients with severe COPD exacerbation and for the establishment of protective or ultraprotective ventilation in patients with ARDS.
- A careful clinical evaluation of patients should be performed to both select the most appropriate ECCO_2_R device in terms of extracorporeal blood flow and consider the potential complications of ECCO_2_R.

## Supplementary Information

**Additional file 1.** Pathophysiology of respiratory acidosis and Pathophysiology of COPD and **Figure S1:** Pathophysiology of COPD exacerbation.

**Additional file 2.** Ongoing research on ECCO_2_R **Table S1:** Ongoing clinical studies on the use of ECCO_2_R in COPD and **Table S2:** Ongoing clinical studies on the use of ECCO_2_R in ARDS.

## Data Availability

Not applicable.
