# Supplementary material for: The use of extracorporeal CO2 removal in acute respiratory failure
Source: Ann Intensive Care. 2021 Mar 11;11:43. doi: 10.1186/s13613-021-00824-6 (PMC7951130; doi:10.1186/s13613-021-00824-6)
Supplement: Supplementary file 1 — Additional file 1. Pathophysiology of respiratory acidosis and Pathophysiology of COPD and Figure S1: Pathophysiology of COPD exacerbation. [file 13613_2021_824_MOESM1_ESM.docx]

**Additional file 1:**

**Pathophysiology of respiratory acidosis**

Respiratory acidosis is defined by acidemia (pH < 7.38) with an increase in PaCO_2_. Hypercapnia is always secondary to alveolar hypoventilation, and hypoxemia is therefore systemic if there is no oxygen supplementation. pH depends on the level of hypercapnia and metabolic compensation (elevation of bicarbonates). The latter is generally delayed from 24 to 48 hours, which makes it possible to distinguish acute hypoventilations (without compensation) from chronic acidosis (with compensation). Normal body CO_2_ production (approximately 220 mL/min) is equivalent to 15,000 mM/day of carbonic acid, compared with values <500 mM/day for all non-respiratory acids treated by the kidneys and intestines. Pulmonary ventilation is adjusted by the respiratory centers according to changes in PaCO_2_, blood pH and PaO_2_, as well as other factors (e.g., exercise, anxiety and alertness).

When the elimination of CO_2_ is insufficient compared to the rate of tissue production, the PaCO_2_ moves to a new state of equilibrium determined by alveolar ventilation and the production of CO_2_. Acutely, the increase in PaCO_2_ increases both [H+] and [HCO3-] ion levels in the blood according to the equilibrium equation of carbonic acid. Thus, the modification of [HCO3-] is simply mediated by the dissociation of H_2_CO_3_ into H+ and HCO_3_- and not by an acute physiological adaptation response. Similarly, the increase in [HCO_3_-] does not "buffer" the increase in [H+]. There is no change in the difference of strong ions and therefore no change in the base excess. Cellular acidosis is always present in cases of respiratory acidosis because CO_2_ accumulates in tissues. If PaCO_2_ remains increased, active compensatory mechanisms are activated, and the difference in strong ions increases to restore the level of [H+] to normal.

The level of CO_2_ in the blood is higher than that of oxygen. CO_2_ is mainly present in blood as bicarbonates and to a lesser extent in dissolved form, whereas functional O_2_ is mainly bound to hemoglobin. However, the dissolved form of CO_2_ has an important role because it ensures the exchange of CO_2_ between the blood and the cell, the place of the production of CO_2_ (Krebs cycle), then, in the lungs, between the blood and the alveolar gas. In addition, small variations in the CO_2_ partial pressure cause significant variations in the level of CO_2_ in the blood, unlike the relationship between the O_2_ partial pressure and the level of O_2_ in the blood. Therefore, in the case of a very high level of carbon dioxide in the blood, extracorporeal CO_2_ purification can take place with relatively low blood flow rates when compared to the blood flow rates required for extracorporeal oxygenation (1).

**Pathophysiology of COPD**

COPD is a common treatable condition characterized by persistent respiratory symptoms and a limitation of exhaled airflow due to airway and/or alveolar abnormalities, and it is usually caused by significant exposure to harmful gases or particles (2). COPD is a major source of morbidity, mortality and major health costs in the western world (3-5). This disease is expected to be the third leading cause of death in the world in 2020 (6). The natural course of the disease consists of periods of clinical stability interrupted by episodes of acute worsening of respiratory symptoms leading to additional treatment, called exacerbations (2). Exacerbations of COPD have a negative impact on health status, hospitalization rate and disease progression. In particular, in-hospital mortality due to exacerbation ranges from 4% to 30% (7), and the 5-year mortality rate after hospitalization is approximately 50% (8).

The limitation of expiratory flow caused by the increased resistance of small airways is the pathophysiological feature of COPD and worsens during exacerbations. The increase in airway resistance is associated with the increase in the expiratory time constant that defines the exponential decrease in lung volume during passive exhalation (9). The direct consequence of a longer time constant is the development of dynamic alveolar hyperinflation, which corresponds to the excessive increase in lung volume at the end of exhalation exceeding the relaxation volume of the respiratory system, generating an intrinsic pressure at the end of expiration (9 , 10).

The acute worsening of dynamic hyperinflation during COPD exacerbations has several detrimental effects on respiratory mechanics, muscular efficiency, alveolar ventilation/perfusion and cardiovascular stability, resulting in gas exchange abnormalities (10-12). In particular, hypercapnia can be severe and correlate with the risk of mortality in the short and long term (11, 13).

The adaptation of the ventilatory muscles to the chronic increase in the resistive, elastic and threshold load can be rapidly exceeded in the event of an increase in dynamic hyperinflation (11), leading to a worsening of hypercapnia. In the most severe cases, a vicious cycle is created, resulting from increased ventilatory motility due to hypoxemia and carbon dioxide (CO_2_) retention, anxiety, fever and sympathetic activation. This mechanism promotes increased pulmonary elastance, dynamic hyperinflation, and muscle fatigue (11, 14). The pathophysiology of COPD exacerbation is shown in **Figure S1**.


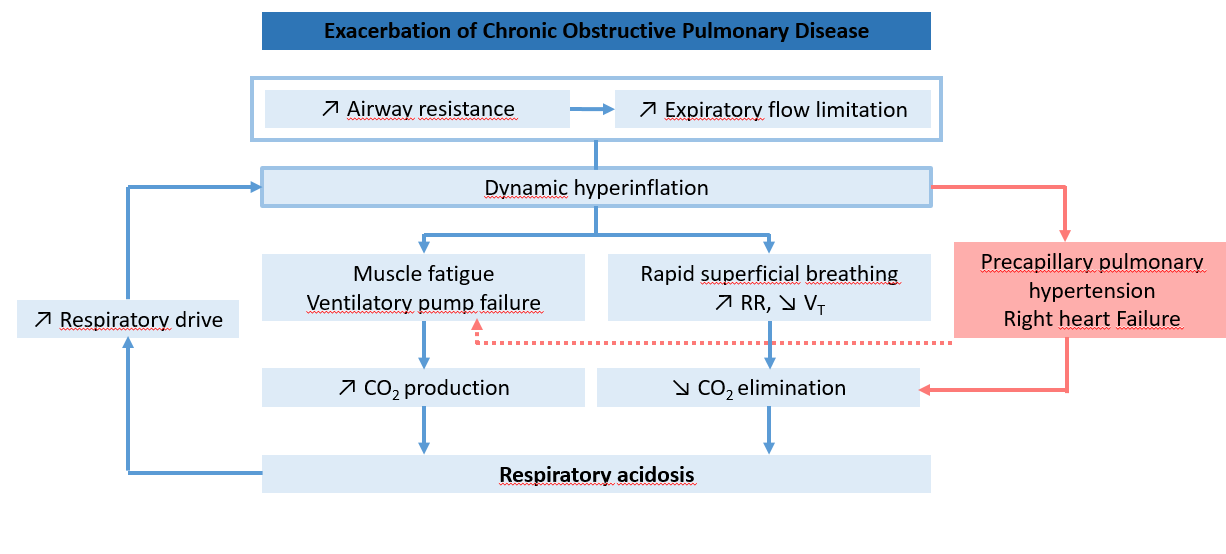


**Figure S1:** Pathophysiology of COPD exacerbation

**Reference:**

1. Diehl JL, Boisrame-Helms J, Chardon-Couteau A, Commereuc M, Augy JL, Sokoloff A, et al. [The role of extracorporeal removal of CO2 (ECCO2R) in the management of respiratory diseases]. Rev Mal Respir. 2017;34(6):598-606.

2. Vogelmeier CF, Criner GJ, Martinez FJ, Anzueto A, Barnes PJ, Bourbeau J, et al. Global Strategy for the Diagnosis, Management, and Prevention of Chronic Obstructive Lung Disease 2017 Report. GOLD Executive Summary. Am J Respir Crit Care Med. 2017;195(5):557-82.

3. Rabe KF, Watz H. Chronic obstructive pulmonary disease. Lancet. 2017;389(10082):1931-40.

4. Khakban A, Sin DD, FitzGerald JM, McManus BM, Ng R, Hollander Z, et al. The Projected Epidemic of Chronic Obstructive Pulmonary Disease Hospitalizations over the Next 15 Years. A Population-based Perspective. Am J Respir Crit Care Med. 2017;195(3):287-91.

5. Collaborators GBDCRD. Global, regional, and national deaths, prevalence, disability-adjusted life years, and years lived with disability for chronic obstructive pulmonary disease and asthma, 1990-2015: a systematic analysis for the Global Burden of Disease Study 2015. Lancet Respir Med. 2017;5(9):691-706.

6. Vestbo J, Hurd SS, Agusti AG, Jones PW, Vogelmeier C, Anzueto A, et al. Global strategy for the diagnosis, management, and prevention of chronic obstructive pulmonary disease: GOLD executive summary. Am J Respir Crit Care Med. 2013;187(4):347-65.

7. Donaldson GC, Wedzicha JA. COPD exacerbations .1: Epidemiology. Thorax. 2006;61(2):164-8.

8. Hoogendoorn M, Hoogenveen RT, Rutten-van Molken MP, Vestbo J, Feenstra TL. Case fatality of COPD exacerbations: a meta-analysis and statistical modelling approach. Eur Respir J. 2011;37(3):508-15.

9. Wedzicha JA, Singh R, Mackay AJ. Acute COPD exacerbations. Clin Chest Med. 2014;35(1):157-63.

10. Laghi F, Goyal A. Auto-PEEP in respiratory failure. Minerva Anestesiol. 2012;78(2):201-21.

11. O'Donnell DE, Parker CM. COPD exacerbations . 3: Pathophysiology. Thorax. 2006;61(4):354-61.

12. Marini JJ. Dynamic hyperinflation and auto-positive end-expiratory pressure: lessons learned over 30 years. Am J Respir Crit Care Med. 2011;184(7):756-62.

13. Barbera JA, Roca J, Ferrer A, Felez MA, Diaz O, Roger N, et al. Mechanisms of worsening gas exchange during acute exacerbations of chronic obstructive pulmonary disease. Eur Respir J. 1997;10(6):1285-91.

14. Loring SH, Garcia-Jacques M, Malhotra A. Pulmonary characteristics in COPD and mechanisms of increased work of breathing. J Appl Physiol (1985). 2009;107(1):309-14.
