# Supplementary material for: The use of extracorporeal CO2 removal in acute respiratory failure
Source: Ann Intensive Care. 2021 Mar 11;11:43. doi: 10.1186/s13613-021-00824-6 (PMC7951130; doi:10.1186/s13613-021-00824-6)
Supplement: Supplementary file 2 — Additional file 2. Ongoing research on ECCO2R Table S1: Ongoing clinical studies on the use of ECCO2R in COPD and Table S2: Ongoing clinical studies on the use of ECCO2R in ARDS. [file 13613_2021_824_MOESM2_ESM.docx]

**Additional file 2:**

**Ongoing research on ECCO_2_R**

Study NCT03584295 is a randomized trial of 202 patients to determine if veno-venous ECCO_2_R can reduce death and/or severe disability to 60 days in patients with severe acute exacerbation of COPD requiring invasive mechanical ventilation. After randomization, patients with acute exacerbation of severe COPD requiring MV will be treated conventionally in the control arm or with vv-ECCO_2_R in the intervention arm to facilitate early extubation. ECCO_2_R will be used in a standard configuration with a double-lumen venous cannula (20-22 Fr) or two small single-lumen venous cannulas (15 to 19 Fr), allowing blood flow between 1 and 2 L/min. The present study suggests that avoiding MV could also significantly improve quality of life, particularly by avoiding tracheostomy and positive pressure ventilation. Improved mobility due to faster recovery also has a major impact on patients' quality of life. The study NCT03255057 is also a randomized trial on 500 patients that plans to evaluate the safety and efficacy of the Hemolung® device as an alternative or a complement to MV in patients requiring respiratory assistance due acute exacerbation of COPD. Eligible patients will be randomized to receive pulmonary support with the Hemolung® ECCO_2_R system combined with MV in the intervention arm or to receive standard MV alone in the control arm. This low-flow ECCO_2_R system uses a 15.5 French dual-lumen catheter inserted percutaneously into the femoral or jugular vein. The aim of this study is to show that using low-flow ECCO_2_R to remove more CO_2_ can avoid or reduce MV duration. The main risks of complications of low-flow ECCO_2_R that will be assessed in this study are associated with central venous catheterization and the need for anticoagulation during treatment. Study NCT02260583 is a physiological pilot study aimed at evaluating the safety and efficacy of this technique for reducing the level of PaCO_2_. Study NCT02259335 is a pilot test whose aim is to assess the possibility of reducing the duration of mechanical ventilation using an extracorporeal CO_2_ purifier to allow early extubation. Study NCT03692117 is observational and aims to assess the clinical efficacy of ECCO_2_R in patients with COPD exacerbation. Finally, in study NCT02965079, the authors aim to conduct an observational study to assess all the patients implanted with an ECCO_2_R device in 10 intensive care units in Paris and its surroundings (APHP, Public Assistance of Hospitals of Paris). These various ongoing clinical studies on the use of ECCO_2_R in COPD and ARDS are summarized in **Table S1 and Table S2,** respectively.


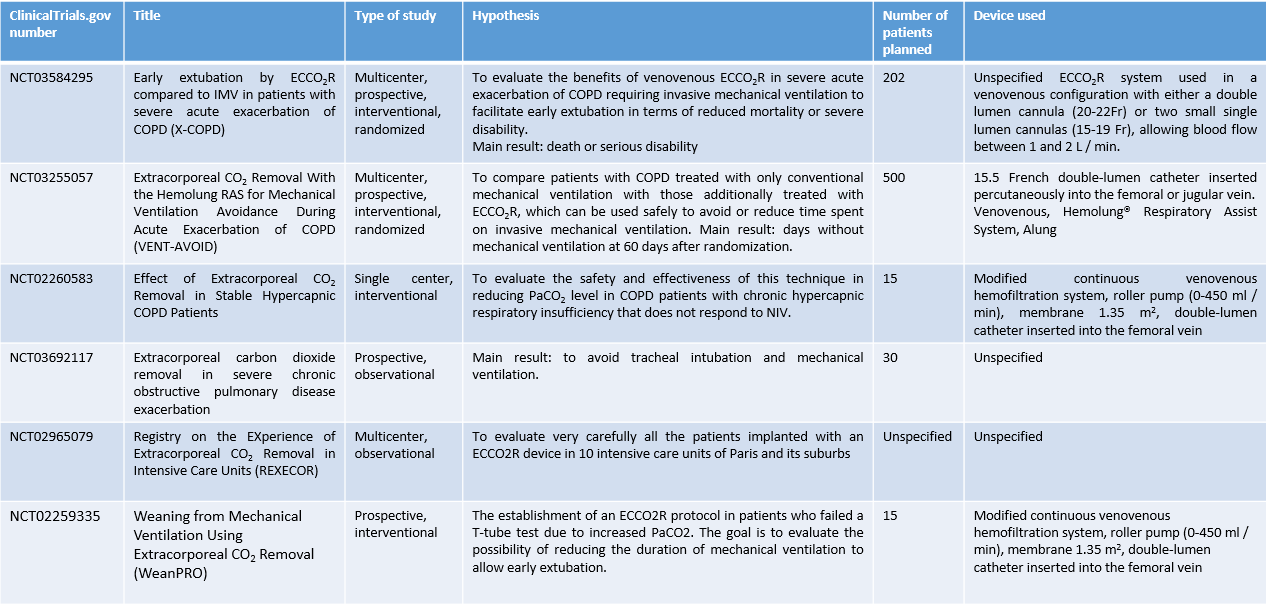


**Table S1:** Ongoing clinical studies on the use of ECCO_2_R in COPD


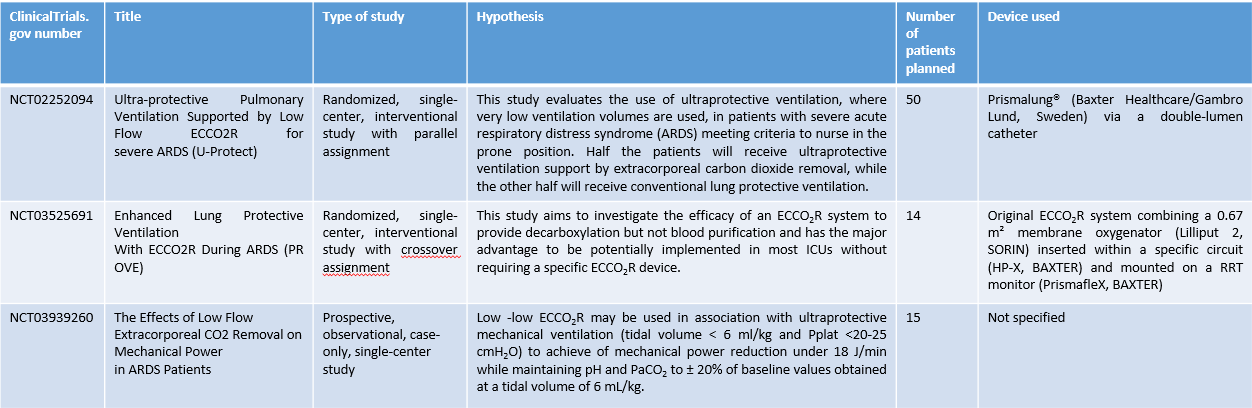


**Table S2:** Ongoing clinical studies on the use of ECCO_2_R in ARDS
